# To Our Readers

**Published:** 1867-12

**Authors:** 


### TO OUR READERS.

What may be said in connection with the pecuniary liabilities of our patrons, we wish read and remembered by all indebted for the JOURNAL, as addressed to each, individually.

With this number, though not the last of the volume, we close the monthly issue for the year 1867; making *twenty-two* consecutive months, since the war, the JOURNAL has been furnished, at a cost of no small amount of labor, and a heavy pecuniary outlay. These things are known, it is true, to every reader of the JOURNAL, should his mind be directed to the subject, but the difficulty is, that each considers his indebtedness a small matter—not sufficient in amount to relieve the total embarrassment of the Editors—and, therefore payment is postponed indefinitely. This, no doubt, is the real cause of delay with many, and is our charitable construction of the conduct of all; for we would hesitate to believe that any member of the profession would receive and read the JOURNAL regularly with the deliberate intention of defrauding. Many, doubtless, fail to pay on account of doubt as to the amount due, and defer inquiries to some convenient time. To all such we say, if the first number was received in 1866, you are due this office $8 00, less the amount you have paid, and if your subscription commenced this year, you owe $4 00, unless you have paid it. When you have received twelve numbers, without payment, and wish to continue, you should send us $8 00, as subscriptions are due in advance.

Remember, friends, *individually*, that when you have received a volume of twelve numbers, you have appropriated four dollars' worth of our property, (by our consent, of course,) the money for which we very much need. If all will determine to send on their dues before Christmas, and carry out that determination, you will not be inconvenienced by so small an amount for each, but will enable us to meet our engagements.

